# Endogenous IL-33 Deficiency Exacerbates Liver Injury and Increases Hepatic Influx of Neutrophils in Acute Murine Viral Hepatitis

**DOI:** 10.1155/2017/1359064

**Published:** 2017-05-04

**Authors:** Virginie Carrière, Muhammad Imran Arshad, Jacques Le Seyec, Benjamin Lefevre, Muhammad Farooq, Aurélien Jan, Christelle Manuel, Laurence Touami-Bernard, Catherine Lucas-Clerc, Valentine Genet, Hugues Gascan, Jean-Philippe Girard, Frédéric Chalmel, Lucie Lamontagne, Claire Piquet-Pellorce, Michel Samson

**Affiliations:** ^1^Institut National de la Santé et de la Recherche Médicale (Inserm), U.1085, Institut de Recherche Santé Environnement et Travail (IRSET), 35043 Rennes, France; ^2^Université de Rennes 1, 35043 Rennes, France; ^3^Structure Fédérative BioSit UMS 3480, CNRS-US18-INSERM, 35043 Rennes, France; ^4^Service de Biochimie CHU Rennes, Université de Rennes 1, Rennes, France; ^5^Institut de Génétique et Développement de Rennes (IGDR), UMR 6290 CNRS, Université de Rennes 1, 35043 Rennes, France; ^6^Institut de Pharmacologie et de Biologie Structurale, Centre National de la Recherche Scientifique (IPBS-CNRS), Université de Toulouse, 31077 Toulouse, France; ^7^Département des Sciences Biologiques, Université du Québec à Montréal, Montréal, Québec, Canada

## Abstract

The alarmin IL-33 has been described to be upregulated in human and murine viral hepatitis. However, the role of endogenous IL-33 in viral hepatitis remains obscure. We aimed to decipher its function by infecting IL-33-deficient mice (IL-33 KO) and their wild-type (WT) littermates with pathogenic mouse hepatitis virus (L2-MHV3). The IL-33 KO mice were more sensitive to L2-MHV3 infection exhibiting higher levels of AST/ALT, higher tissue damage, significant weight loss, and earlier death. An increased depletion of B and T lymphocytes, NKT cells, dendritic cells, and macrophages was observed 48 h postinfection (PI) in IL-33 KO mice than that in WT mice. In contrast, a massive influx of neutrophils was observed in IL-33 KO mice at 48 h PI. A transcriptomic study of inflammatory and cell-signaling genes revealed the overexpression of IL-6, TNF*α*, and several chemokines involved in recruitment/activation of neutrophils (CXCL2, CXCL5, CCL2, and CCL6) at 72 h PI in IL-33 KO mice. However, the IFN*γ* was strongly induced in WT mice with less profound expression in IL-33 KO mice demonstrating that endogenous IL-33 regulated IFN*γ* expression during L2-MHV3 hepatitis. In conclusion, we demonstrated that endogenous IL-33 had multifaceted immunoregulatory effect during viral hepatitis via induction of IFN*γ*, survival effect on immune cells, and infiltration of neutrophils in the liver.

## 1. Introduction

The cytokine IL-33 is the 11th member of the IL-1 family and is designated as IL-1F11. It is now described as a DAMP (damage-associated molecular patterns) or “alarmin” molecule that is normally restrained to the nuclear compartment where it could act as a nuclear factor-regulating gene expression [1] but is released in case of pathogen aggression or injury to alert the immune system [2, 3]. Once released from the cells, IL-33 mediates its function through interaction with its specific receptor a dimer of ST2 (IL-1 receptor-like 1) and IL-1RacP (IL-1 receptor accessory protein) [4]. IL-33 has been described to be constitutively expressed in cell-lining tissues like endothelial cells, epithelial cells, keratinocytes, and fibroblasts but could also be induced in other cells like hepatocytes [5]. IL-33 released into the extracellular space after cell damage could be active as a full-length form (~33 kDa), but proteases have been shown to regulate IL-33 activity. For instance, in case of apoptosis, caspases 3 and 7 cleaved IL-33 generating two biologically inactive form of IL-33 [6], whereas during inflammation, neutrophil serine proteases, cathepsin G, and elastase were found to process IL-33 into mature forms of ~20 kDa with increased biological activity (by 10-fold) [7]. The ST2 receptor is expressed in both innate and adaptive immune cells and is predominantly associated with type 2 immune response [4]. The ST2-expressing cells, a target of IL-33, are macrophages, dendritic cells, mast cells, eosinophils, neutrophils, recently described nuocytes, or innate lymphoid cells such as ILC2 [8], Th2 cells, but could also be transient on the surface of Th1 cells [9] and antiviral CD8^+^ T cells [10].

The IL-33/ST2 axis has been described in various pathologies and affected organs, and it induced deleterious or protective effects [11, 12] depending upon the immune mediators and inflammatory milieu. In liver pathology, we and others reported increased level of serum IL-33 and ST2 not only in acute and chronic hepatic failure in humans but also in patients chronically affected with hepatitis C and hepatitis B viruses (HCV and HBV) in correlation with liver damage [13–16]. Upregulated expression of IL-33 was reported in a murine model of T cell-mediated hepatitis mice infected with hepatotropic lymphocytic choriomeningitis virus (LCMV) [10, 17]. In LCMV hepatitis, IL-33 induced hepatoprotective by promoting innate IFN*γ* production and modulating dendritic cells response, driving an antiviral CD8^+^ T cell response [10, 17]. In another mouse model of adenoviral hepatitis, the expression of IL-33 and ST2 was observed in the liver in the first week of infection, and it attenuated liver injury via an increase in a number of regulatory T cells (Treg) and decrease in a number of macrophages, dendritic cells, and NK cells in the liver [18]. However, there is a paucity of data regarding endogenous IL-33 deficiency on immunomodulatory effect in viral hepatitis. The present study investigated the role of IL-33 in the natural model of acute hepatitis in mice induced by specific mouse hepatitis coronavirus (MHV3). The hepatotropic and pathogenic MHV3 strain (L2-MHV3) induced severe fulminant hepatitis in mice and their death within 3–5 days postinfection [19], mimicking a human viral hepatitis. We previously demonstrated L2-MHV3-induced overexpression of IL-33 in liver sinusoidal endothelial cells, vascular endothelial cells, and hepatocytes [20]. Here, infecting wild-type (WT) and IL-33-deficient mice (IL-33 KO) with L2-MHV3, we showed that the alarmin IL-33 ameliorated the L2-MHV3-mediated liver injury through the regulation of IFN*γ* expression, survival effect on immune cells, and neutrophil infiltration in viral hepatitis.

## 2. Materials and Methods

### 2.1. Animals

Eight- to ten-week-old wild-type (WT) C57Bl/6 (Janvier, Le Genest-sur-isle, France) and littermate IL-33 knockout (KO) C57Bl/6 mice (matched for age and sex) were provided by Dr. Jean-Philippe Girard [21]. The mice used in the study were certified as MHV3-free by the manufacturer, and they were housed under HEPA-filtered air (Forma Scientific, Marietta, OH) in the local BSL3 animal facility. The study was conducted in compliance with the French laws and the institution's guidelines for animal welfare, and the protocol was approved by the Committee on the Ethics of Animal Experiments of the French government (agreement of M. Samson number 3596). All efforts were made to minimize suffering and pain of the animals.

### 2.2. In Vivo Treatment Protocol

For L2-MHV3 infection in mice, the C57BL/6 mice were infected by the ip route with 10^3^ 50% tissue culture infective dose (TCID 50) of pathogenic L2-MHV3 strain as previously described [22]. Mock-infected or uninfected control mice received a similar volume of PBS. Mice were followed up twice a day for weight loss and temperature measurement. After 48 h and 72 h of infection, the mice were euthanized by cerebral dislocation before liver and blood sampling. The mice were followed up for 7 days for survival curve analysis. For the infection of primary mouse hepatocytes (PMH) by L2-MHV3, an MOI (multiplicity of infection) of 1 was used.

### 2.3. Primary Mouse Hepatocyte (PMH) Isolation and Culture

Primary mouse hepatocytes were isolated according to retrograde perfusion approach [23]. Briefly, anesthetized C57/BL6 mice were perfused through the inferior vena cava with Hepes buffer added with Liberase (2.3 mg/ml Roche®) and CaCl_2_ to start in vivo hepatocyte dissociation. The liver was then transferred to the petri dish with Leibovitz medium, and liver capsule was broken to release hepatocytes before filtration, washed with Hepes buffer, and transferred to Williams' E medium. Alive hepatocytes were isolated on 35% gradient percoll before seeding on a collagen-covered dish in Williams' E medium added with 2 mM glutamine, 10 IU/ml penicillin, 10 *μ*g/ml streptomycin, 10% SVF, and 5 *μ*g/ml insulin. After 6 h, attached hepatocytes were cultured in maintenance medium and Williams' E medium in which SVF was replaced by 1 mg/ml bovine serum albumin (Eurobio) and 0.1 *μ*M dexamethasone (Sigma).

### 2.4. Histopathological, Biochemical, and Immunohistochemical Analyses

The histopathological (hematoxylin and eosin (H&E) staining) and level of liver transaminases (ALT/AST) in serum were performed as described earlier. Immunolocalisation of IL-33 was performed by immunohistochemical staining using primary antibody anti-mouse-IL-33 (goat IgG, R&D Systems). Leucocytes were stained with anti-mouse CD45 antibody (clone 30-F11, BD Pharmingen), macrophages with anti-mouse F4/80 antibody (clone BM8, eBioscience), and neutrophils, after antigen retrieval step (citrate buffer pH 6), with anti-mouse Ly6G antibody (clone 1A8, BD Bioscience). Then, secondary HRP-conjugated dedicated specific antibodies (Dako, USA) were used followed by hematoxylin counterstaining in a Ventana machine (Ventana Medical Systems Inc., USA). The counting of stained positive cells was carried in at least 3 different microscopic fields of 1 mm^2^ surface area by tissues on at least 3 different tissues by conditions by using image analysis software (NDP, view, Hamamatsu).

### 2.5. RNA Isolation and RT-qPCR

The protocol and conditions for RNA extraction, RT-PCR, and qPCR were similar as reported earlier by our laboratory [5, 24]. The specific primers used are presented in Supplementary Table 1 available online at https://doi.org/10.1155/2017/1359064. The relative gene expression was normalized against 18S gene expression. The mean expression of control mice in each treatment group served as a reference for mRNA expression (control mRNA level was arbitrarily taken as 1). For a wide transcriptomic study, the 1 *μ*g RNA was obtained after pooling equal amount of RNA from different mouse liver from the same conditions. After RT-PCR, qPCR was made with 344 oligo pair bank and normalized against 18S gene expression. The PBS-treated WT mice served as a reference and their mRNA expression was arbitrarily considered as 1. Data processing and gene filtration was carried out using the AMEN suite of tools [25]. Briefly, fold-change values were log2 transformed. Next, 284 genes showing at least a 2-fold change in at least one pairwise comparison were selected. The resulting genes were classified into 13 expression patterns (termed P1–P13) using the k-means algorithm bioinformatics analysis.

### 2.6. Western Blot

A piece of fast-frozen mouse liver was homogenized in RIPA buffer by Ultra-Turrax® and left on the ice during 40 min, vortexed regularly before a centrifugation of 10000*g* at 4°C. The supernatant was then recovered before Bradford dosage. 50 *μ*g of total protein extract was then loaded on polyacrylamide gel, transferred to a nitrocellulose membrane before incubation overnight in IL-33 antibody (goat Ab, R&D) in TBS 5% milk and 1 hour in secondary HRP-conjugated rabbit anti-goat antibody (Dako, USA), and followed by a revelation with ECL (Pierce).

### 2.7. Cytokine Dosage

The levels of IFN*γ*, IL-2, IL-6, IL-10, and IL-17A were measured in at least 5 mouse sera by condition according to the protocol and instructions of the manufacturer (DIAplex murine 5plex (Diaclone®).

### 2.8. Flow Cytometry Analysis

Twelve mice were grouped as WT versus IL-33 KO, and they were investigated at the time point of infection control versus 48 h postinfection (PI) versus 72 h PI by flow cytometry. After the sacrifice, the liver was perfused with PBS via the portal vein and then passed through a 60 *μ*m cell strainer to dissociate the cells (BD Biosciences). Parenchymal cells were removed by decantation for 1 h. The cells in suspension were recovered by centrifugation and then resuspended in 35% isotonic Percoll (GE Healthcare Life Sciences). Liver leukocytes (LL) were pelleted by centrifugation for 25 min at 2600 rpm at 25°C. After lysis of red blood cells, LL was resuspended in staining buffer PBS 2% FBS (Perbio) and counted. The LL was firstly stained with orange live/dead cells staining (Life Technologies) in order to discriminate between live/dead cells. The cells were then incubated with appropriate dilutions of various combinations of the following fluorochrome-conjugated antibodies/reagents: CD1d tetramer-*α*-GalCer-APC (provided by Maria Leite-De-Moraes), CD11c-APC (clone HL3), anti-Gr-1-FITC (clone RB6-8C5), anti-CD11b-PE Cy™7 (clone M1/70), anti-CD69-PE (clone H1.2F3) or anti-CD3ε-Pacific Blue (clone 500A2), anti-NK1.1-PerCP-Cy-5.5 (clone PK136), and anti-CD8*α*-APC-Cy7 (clone 53–6.7) antibodies, all purchased from BD PharMingen. The cells were then washed; fixed in PBS containing 2% FCS, 0.01 M sodium azide, and 2% formaldehyde; and analyzed on FACSAriaTM II flow cytometer using BD FACSDiva software (BD Bioscience), and the data were processed using CXP software (Beckman Coulter). Dead cells and doublet cells were excluded on the basis of forward and side scatter.

### 2.9. Statistical Analysis

Each dot on the graph represents results obtained for each mouse individually. The Mann-Whitney *U* test using GraphPad Prism5 software was done to compare the parameters between WT and IL-33 KO mice in in vivo studies. # represents a significant difference with WT PBS condition with ^#^*p* < 0.05, ^##^*p* < 0.01, and ^###^*p* < 0.001. ^∗^ represents a significant difference between WT and IL-33 KO conditions at the same time PI with ^∗^*p* < 0.05, ^∗∗^*p* < 0.01, and ^∗∗∗^*p* < 0.001. $ represents significant difference between two conditions in the same background mice with ^$^*p* < 0.05, ^$$^*p* < 0.01, and ^$$$^*p* < 0.001.

## 3. Results

### 3.1. The Pathogenic L2-MHV3 Infection Upregulated Transcript and Active Form of IL-33 in the Liver of WT Mice

**Figure 1:**
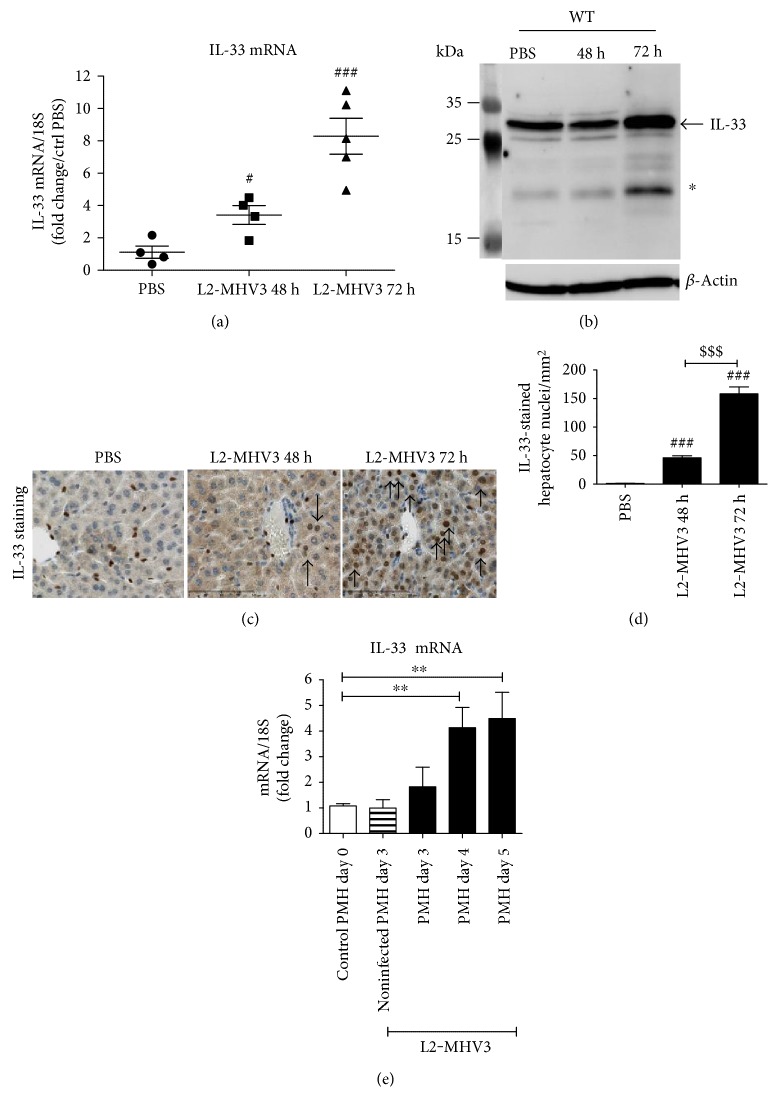
Transcript and protein expression of IL-33 in the liver of L2-MHV3-infected WT mice. (a) Quantification of IL-33mRNA expression by RT-qPCR in the liver of WT control mice (PBS) and 48 h PI and 72 h PI L2-MHV3 infection. The results were normalized to 18S expression and expressed as a fold change compared to the noninfected (PBS) condition. (b) Total liver protein of WT mice control (PBS) or from WT L2-MHV3-infected mice 48 h or 72 h PI were analyzed by western blotting for IL-33 expression. (c) IL-33 immunostaining of paraffin-embedded mouse liver control (PBS) or infected with L2-MHV3 for 48 h or 72 h (arrows indicate IL-33-stained hepatocyte nuclei). (d) Counting of IL-33-stained nuclei in paraffin-embedded liver immunostained for IL-33 from WT mice control (PBS) or infected with L2-MHV3 for 48 h or 72 h. (e) Quantification of IL-33 mRNA induction by RT-qPCR in primary mouse hepatocytes (PMH) infected in vitro with L2-MHV3 (MOI 1). The basal expression of IL-33 in PMH before infection (day 0) was used as a control and arbitrarily considered as 1 AU (arbitrary unit) which served as a reference for fold change in other conditions. Statistical analyses were performed according to Student's *t*-test. ^∗^*p* < 0.05; ^∗∗^*p* < 0.001.

Infecting C57BL/6 wild-type (WT) mice with the natural pathogenic mouse hepatitis virus (L2-MHV3) significantly upregulated the local mRNA expression of IL-33 in the liver with a 3.4- and 8.3-fold increase at 48 h and 72 h PI compared to that of PBS control (Figure 1(a)). At protein level, we demonstrated that the upregulated transcript expression was associated with an increased expression of the full-length IL-33 protein in total liver extract of WT mice (Figure 1(b), indicated with an arrow) and a short form of IL-33 with a molecular weight of ~20 kDa (Figure 1(b), indicated with a star) especially at 72 h PI. The immunostaining of liver tissues revealed that IL-33 was initially expressed only in the sinusoidal and vascular endothelial cells in the liver of control WT mice (Figure 1(c), brown staining in PBS condition) and induced in the nuclei of hepatocytes during L2-MHV3 infection (48 h and 72 h PI) (Figure 1(c), arrow). The number of IL-33-expressing hepatocytes was significantly increased following L2-MHV3 infection with a higher number at 72 h PI (Figure 1(d)). To ascertain the direct induction of IL-33 in hepatocytes by L2-MHV3, freshly isolated primary mouse hepatocytes (PMH) were infected in vitro with L2-MHV3 for 7 days. The cytopathic effect (syncytia and lysis of monolayer cells) appeared from day 3 PI (data not shown) and a significant increase of mRNA expression of IL-33 was observed at day 4 and day 5 of L2-MHV3 infection. This demonstrated that in vitro L2-MHV3 was sufficient to induce IL-33 expression in hepatocytes (Figure 1(e)).

### 3.2. Endogenous IL-33 has a Protective Role during L2-MHV3-Induced Acute Hepatitis in Mice

**Figure 2:**
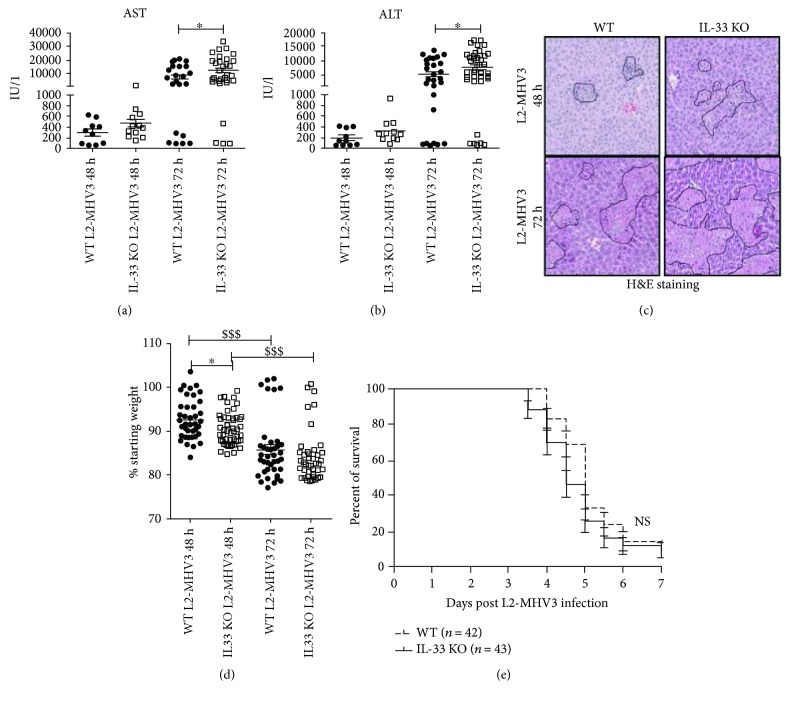
Liver injury and survival curve of WT and IL-33 KO mice following L2-MHV3 infection. (a) Comparison of AST and (b) ALT level in sera of WT or IL-33 KO mice infected with L2-MHV3 at 48 h (*n* = 10 WT versus *n* = 12 IL-33 KO) or 72 h (*n* = 28 WT versus *n* = 39 IL-33 KO). (c) Liver sections of WT and IL-33 KO mice at 48 h and 72 h L2-MHV3 PI stained with H&E for histopathology to visualize zone of liver injury (dotted line in black). (d) Weight loss quantification in WT and IL-33 KO after 48 h (*n* = 43 WT versus 47 IL-33 KO) or 72 h (*n* = 39 WT versus *n* = 41 IL-33 KO) of L2-MHV3 infection. (e) Survival curve of WT (*n* = 42) or IL-33 KO (*n* = 43) mice after L2-MHV3 infection. Statistical analyses were performed according to the Mann-Whitney *U* test. ^∗^*p* < 0.05; ^∗∗∗^*p* < 0.0001. ^∗^ symbolizes the significant difference between WT and IL-33 KO mice, whereas $ symbolizes the significant difference between two conditions in the same background mice.

We next studied the effect of depletion of endogenous IL-33 on L2-MHV3 mediated viral hepatitis by using IL-33-deficient mice (IL-33 KO). The liver injury markers (AST and ALT level) showed a significantly higher level of AST and ALT at 72 h PI in IL-33 KO mice than that in WT mice (Figures 2(a) and 2(b)). Concomitantly, the liver histology revealed increased necrotic area in IL-33 KO mice livers with higher infiltrate cells as compared to that in WT mice liver at 48 and 72 h PI (Figure 2(c)). The L2-MHV3 infection significantly induced weight loss of both WT and IL-33 KO mice at 48 h and 72 h PI but with a greater loss as soon as 48 h PI in IL-33 KO mice (Figure 2(d)). The survival curve analysis revealed that the L2-MHV3-infected IL-33 KO mice started dying earlier between 3 and 6 days PI than the WT mice (median survival 4.5 versus 5 days) but in a nonsignificant manner on the 7 days of viral infection (Figure 2(e)).

### 3.3. The Amplification of MHV3 or Innate Immune TLR Expression was Not Altered by Genetic Deficiency of IL-33 in Mice

**Figure 3:**
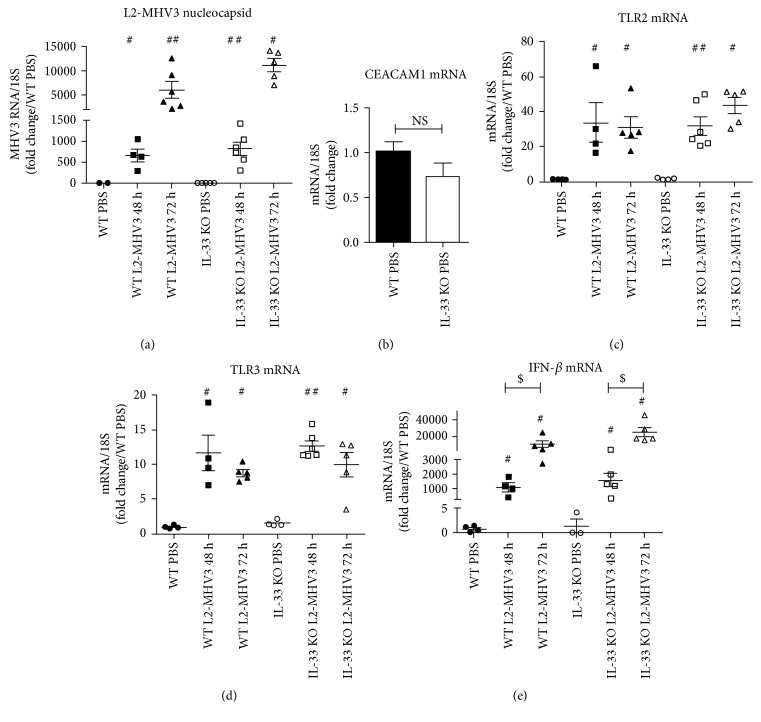
Comparison of L2-MHV3 viral nucleocapsid, mRNA expression of viral recognition receptors, and IFN-*β* expression in WT and IL-33 KO mice. Total liver RNA was extracted from WT and IL-33 KO mice control (PBS) or L2-MHV3-infected mice (48 h and 72 h PI) and tested by RT-qPCR for (a) L2-MHV3 nucleocapsid viral RNA expression, (b) CEACAM1 mRNA, (c) TLR2 mRNA, (d) TLR3 mRNA, and (e) IFN-*β* mRNA. The mean expression of the WT PBS mice was used as a control and arbitrarily considered as 1 AU (arbitrary unit) which served as a reference for fold change in other conditions. Statistical analyses were done according to the Mann-Whitney *U* test. # represents significant difference with WT PBS mice, ^∗^ represents significant difference between WT and IL-33 KO conditions, and $ represents significant difference between two conditions in the same background mice. ^#^*p* < 0.05; ^##^*p* < 0.01; and ^###^*p* < 0.001.

As the IL-33 KO mice were more sensitive to L2-MHV3 infection, we measured the viral amplification in the liver by quantification of viral nucleocapsid coding RNA. The nucleocapsid viral RNA of L2-MHV3 increased exponentially with the time of infection, and L2-MHV3-infected IL-33 KO mice exhibited nonsignificantly but slightly higher viral RNA of L2-MHV3 nucleocapsid compared to WT at 48 h PI (817 versus 655 arbitrary unit, respectively) or at 72 h PI (11176 versus 6058 arbitrary unit, respectively) (Figure 3(a)). At basal level, the mRNA expression of CEACAM-1, the specific MHV3 attachment receptor in the liver, revealed that IL-33 KO control mice presented nonsignificantly decreased transcript level of CEACAM-1 compared to WT mice (Figure 3(b)). The mRNA expression of innate immune receptors, that is, TLR2 and TLR3, the two TLRs described to sense the MHV3 in the liver, exhibited a significantly increased expression at 48 h PI and 72 h PI compared to PBS control in WT and IL-33 KO mice without any significant difference between them (Figures 3(b) and 3(c)). Similarly, the transcript level of antiviral IFN-beta (IFN-*β*) increased exponentially following L2-MHV3-induced acute hepatitis (48 h and 72 h PI) compared to PBS control with a comparable expression in WT and IL-33 KO mice (Figure 3(e)). These data suggest that IL-33 deficiency in mice may not affect the amplification of virus or transcriptional regulation of TLR following L2-MHV3 infection in the host cells.

### 3.4. The Deficiency of IL-33 Led to Early Depletion of Infiltrated Lymphoid Cells in the Liver during L2-MHV3-Induced Acute Hepatitis

**Figure 4:**
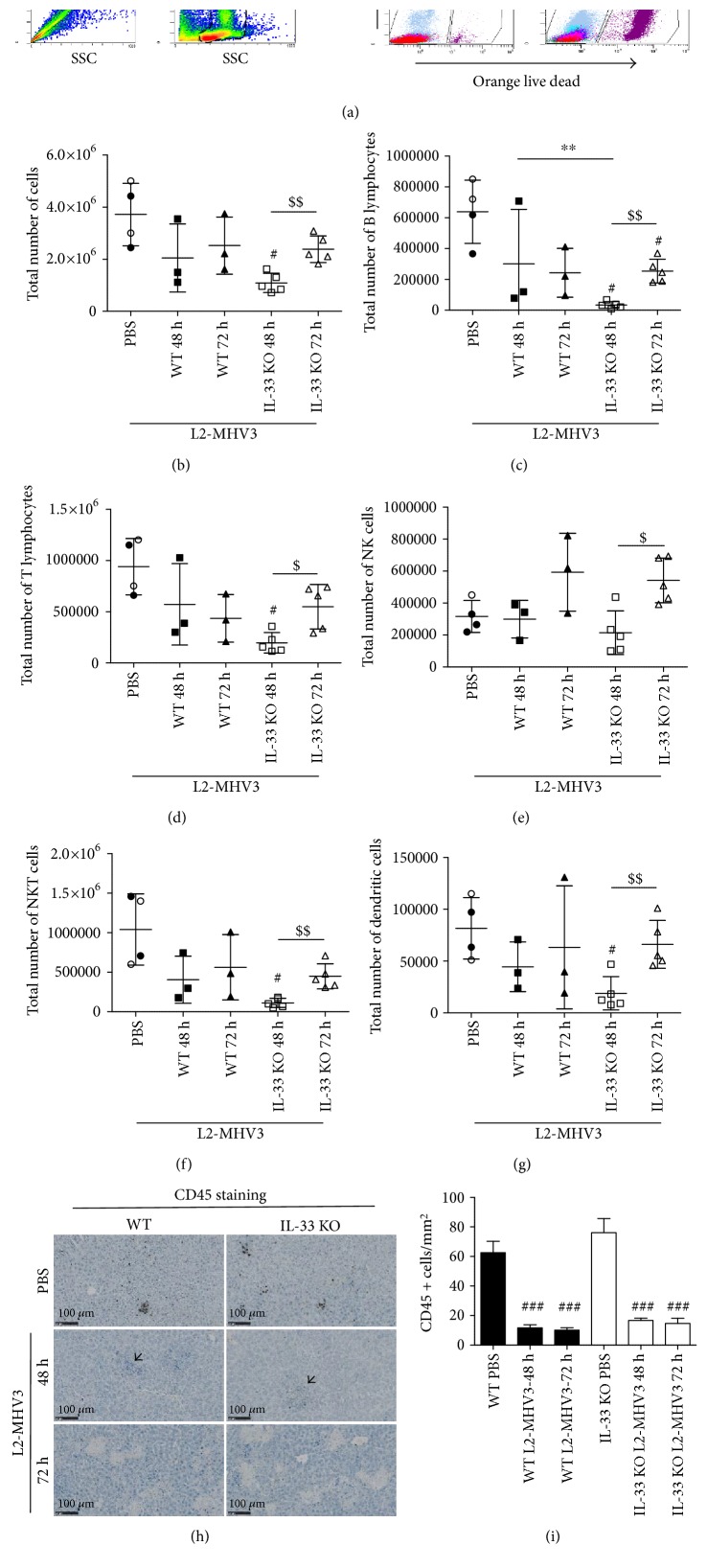
Flow cytometry analysis and immunohistochemistry of liver-infiltrated lymphoid cells in the WT and IL-33 KO mice following L2-MHV3-induced hepatitis. (a) The strategy of gating alive cells after SSC/SSC and FSC/SSC gating and orange live-dead staining. (b) Total number of cells, (c) B lymphocytes, (d) T lymphocytes, (e) NK cells, (f) NKT cells, and (g) dendritic cells were quantified in the liver of WT and IL-33 KO mice in control PBS condition (black dot for WT PBS and clear dot for IL-33 KO PBS) and after 48 h and 72 h L2-MHV3 infection. (h) CD45 immunostaining of paraffin-embedded mouse liver control (PBS) or infected with L2-MHV3 for 48 h or 72 h. Arrowhead highlights inflammatory foci unstained with CD45 antibody. (i) Quantification of CD45-stained cells on 4 areas of 1 mm^2^ by tissues and at least 3 different tissues by condition. Statistical analyses were done according to the Mann-Whitney *U* test. # represents significant difference with WT PBS condition, ^∗^ represents significant difference between WT and IL-33 KO conditions, and $ represents significant difference between two conditions in the same background mice. ^#^*p* < 0.05; ^##^*p* < 0.01; and ^###^*p* < 0.001.

To delineate the role of IL-33 in the regulation of infiltrated cells during L2-MHV3 infection, we investigated the effect of IL-33 ablation on resident immune cells infiltration in the liver following L2-MHV3 infection by flow cytometry. The isolated infiltrate cells were gated to select single and complete cells; the live cells were then discriminated according to orange live-dead staining (Figure 4(a)). After 72 h PI in WT mice or IL-33 KO mice liver, a large number of dead cells were observed (Figure 4(a) right panel). The total number of infiltrate cells in the liver was not varied between WT and IL-33 KO control mice (Figure 4(b), black spot for WT and empty plot for IL-33 KO). The total number of infiltrated cells in WT and IL-33 KO mice at 48 h and 72 h of L2-MHV3 PI was reduced compared to that in control mice (Figure 4(b)). A significant decrease in a total number of infiltrating cells at 48 h PI was observed in IL-33 KO only with a recolonization of cells at 72 h PI (Figure 4(b)). The deficiency of IL-33 led to decrease in number of liver-infiltrated B lymphocytes (CD19^+^ CD8^−^) (Figure 4(c)), T lymphocytes (CD3^+^ NK1.1^−^) (Figure 4(d)), NKT cells (CD3^+^ NK1.1^+^) (Figure 4(f)), and dendritic cells (GR1^−^ CD11b^−^ CD11c^+^) (Figure 4(g)) but not of NK cells (CD3^−^ NK1.1^+^) (Figure 4(e)) at 48 h PI in comparison to that in WT mice. These infiltrated cells (B lymphocytes, T lymphocytes, NK cells, NKT cells, and DCs) were significantly higher at 72 h PI than 48 h PI in the liver of IL-33 KO mice only to reach back the level of WT at 72 h PI. The immunostaining of CD45-positive leukocytes in the liver confirmed the significant decrease in the number of these infiltrated cells at 48 h and 72 h PI in the WT and IL-33 KO mice (Figures 4(h) and 4(i)). Some inflammatory foci were nonetheless visible within the tissues at 48 h PI (indicated with arrowhead) but were not stained with the CD45 antibody.

### 3.5. MHV3 Infection Led to a Total Disappearance of Liver-Infiltrated Macrophages in both Mice but a Massive Influx of Neutrophils in IL-33 KO Mice at 48 h PI

**Figure 5:**
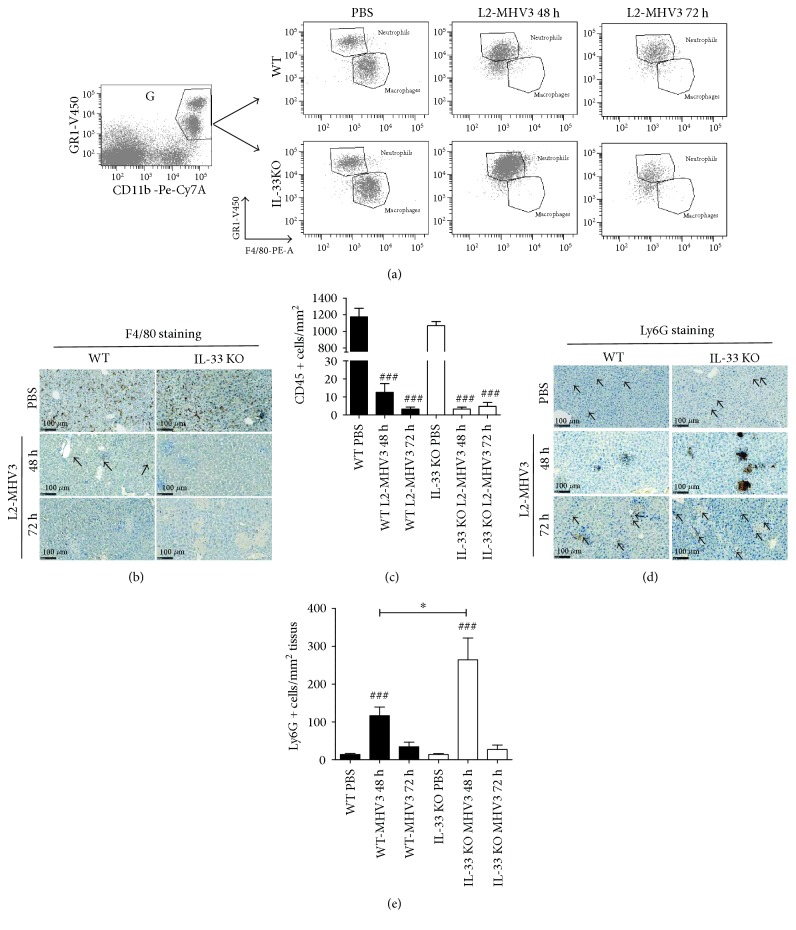
Flow cytometry analysis and immunohistochemistry of liver-infiltrated myeloid cells in the WT and IL-33 KO following L2-MHV3-induced hepatitis. (a) Representative dot-plot of gated myeloid cells according to GR1, CD11b, and F4/80 staining. Macrophages are considered as GR1^interm^/CD11b^+^/F4/80^+^ and neutrophils as GR1^+^/CD11b^+^/F4/80^low^-stained cells. Presented dot plots are representative results observed in 5 different mice by conditions. (b) F4/80 immunostaining of paraffin-embedded mouse liver control (PBS) or infected with L2-MHV3 for 48 h or 72 h. (c) Quantification of F4/80-stained cells on 4 areas of 0.1 mm^2^ by tissues and at least 3 different tissues by condition. (d) Ly6G immunostaining of paraffin-embedded mouse liver control (PBS) or infected with L2-MHV3 for 48 h or 72 h. (e) Quantification of Ly6G-stained cells (neutrophils) on 2 areas of 2 mm^2^ by tissues and at least 3 different tissues by condition. Statistical analyses were done according to the Mann-Whitney *U* test. # represents significant difference with WT PBS condition, ^∗^ represents significant difference between WT and IL-33 KO conditions, and $ represents significant difference between two conditions in the same background mice. ^#^*p* < 0.05; ^##^*p* < 0.01; and ^###^*p* < 0.001.

Analysis of myeloid population by flow cytometry, based on GR1, CD11b, and F4/80 staining (Figure 5(a)) on alive complete cells (as previously described), revealed a drastic fall in the number of macrophages (CD11b^+^, GR1^low^, and F4/80^+^) in WT and IL-33 KO mice at 48 h and 72 h PI, in correlation with total disappearance of F4/80 staining in immunohistochemistry (Figures 5(b) and 5(c)). An increased number of infiltrated neutrophils (CD11b^+^, GR1^+^) at 48 h PI were observed in an exacerbated and significant manner in IL-33 KO mice compared to WT mice (Figure 5(a)). This massive influx of neutrophils was confirmed by a strong Ly6G staining of accumulated infiltrated cells in the liver of IL-33 KO mice by immunohistochemistry (Figures 5(d) and 5(e)).

In conclusion, the absence of endogenous IL-33 significantly affected the liver infiltration of B lymphocytes, T lymphocytes, NK cells, NKT cells, DCs, and macrophages at early onset of L2-MHV3 viral infection (48 h PI) with a recolonization phenomenon at 72 h PI (except for macrophages). In contrast, IL-33 deficiency in mice led to an increase in a number of neutrophils at 48 h of the L2-MHV3 challenge than that in WT mice suggesting an early effect of IL-33 in the recruitment of these cells in the liver.

### 3.6. The Chemokines Associated with Neutrophils Recruitment and Activation Were Upregulated in IL-33 KO Mice

**Figure 6:**
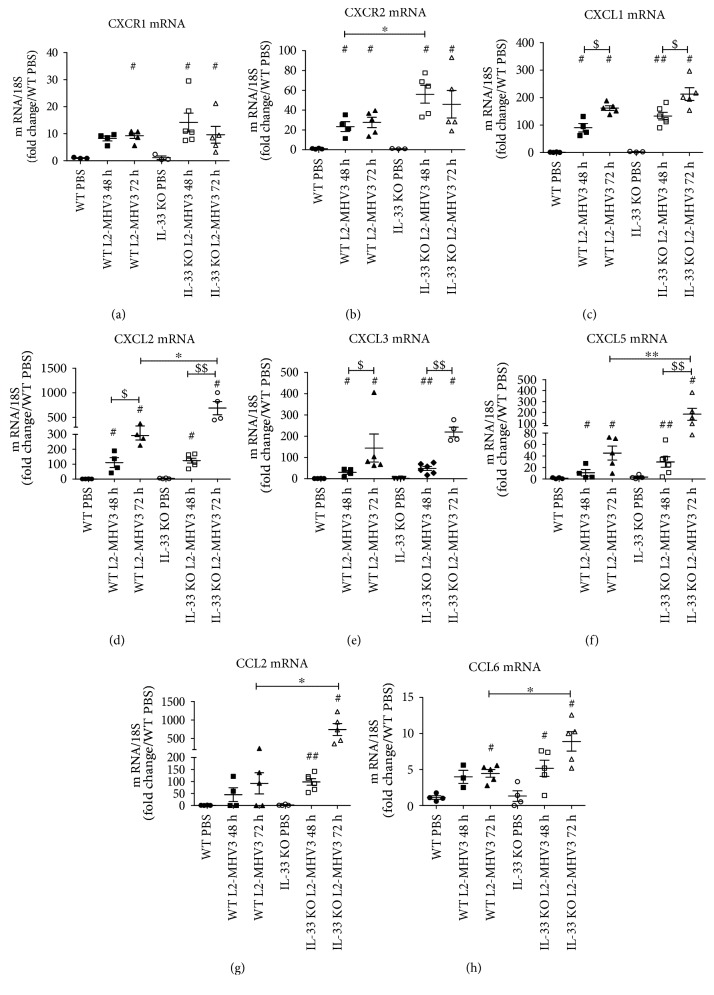
Comparative mRNA expression of neutrophil recruitment-associated chemokines in L2-MHV3-infected WT and IL-33 KO mice. Total liver RNA was extracted from WT or IL-33 KO mice and control (PBS) or L2-MHV3-infected mice (48 h and 72 h PI) and tested by RT-qPCR for (a) CXCR1, (b) CXCR2, (c) CXCL1, (d) CXCL2, (e) CXCL3, (f) CXCL5, (g) CCL2, and (h) CCL6. The mean expression of the WT PBS mice was used as a control and arbitrarily considered as 1 AU (arbitrary unit) which served as a reference for fold change in other conditions. Statistical analyses were done according to the Mann-Whitney *U* test. # represents significant difference with WT PBS mice, ^∗^ represents significant difference between WT and IL-33 KO mice, and $ represents significant difference between two conditions in the same background mice. ^#^*p* < 0.05; ^##^*p* < 0.01; and ^###^*p* < 0.001.

In order to decipher the differential gene expression between the two mouse strains during L2-MHV3-induced hepatitis, transcripts for each condition (a pool of 5 different mice per condition) (WT PBS, WT 48 h L2-MHV3, and WT 72 h L2-MHV3; IL-33 KO PBS, IL-33 KO 48 h L2-MHV3, and IL-33 KO 72 h L2-MHV3) were quantified with a wide transcriptomic bank targeting 344 genes involved in inflammation/chemotaxis and cell signaling. The resulting genes were classified into 13 expression patterns (termed P1–P13) using the k-means algorithm bioinformatics analysis (Supplementary Figure 1 and Supplementary Table 2). Among the “over-induced” genes in L2-MHV3-infected IL-33 KO mice, a network of genes linked to neutrophils, their recruitment and activation were upregulated (Supplementary Table 3). The liver transcriptional expression of several genes of interest was quantified at individual mouse level. First, an increased expression of CXCR1 (Figure 6(a)) and more specifically CXCR2 (Figure 6(b)), receptors expressed by neutrophils, was observed during the time of infection in both mice type with significant upregulation at 48 h PI in IL-33 KO mice compared to WT in accordance with increased number of neutrophils as observed previously (Figures 5(a), 5(d), and 5(e)).

The liver transcriptional expressions of CXCL1, CXCL2, CXCL3, CXCL5, CCL2, and CCL6 significantly increased in WT and IL-33 KO mice during L2-MHV3-induced hepatitis compared to those in control PBS-injected mice as soon as 48 h PI and at 72 h PI (Figures 6(c), 6(d), 6(e), 6(f), 6(g), and 6(h)). Moreover, at 72 h of L2-MHV3 PI, the expressions of CXCL2, CXCL5, CCL2, and CCL6 chemokines were significantly upregulated in IL-33 KO mice in comparison to those in WT mice. Thus, IL-33 affected or controlled the recruitment of neutrophils in the liver following L2-MHV3 acute hepatitis.

### 3.7. IL-33 KO Mice Exhibited More IL-6 and TNF*α* but Less IFN*γ* Expression during L2-MHV3-Induced Acute Hepatitis

**Figure 7:**
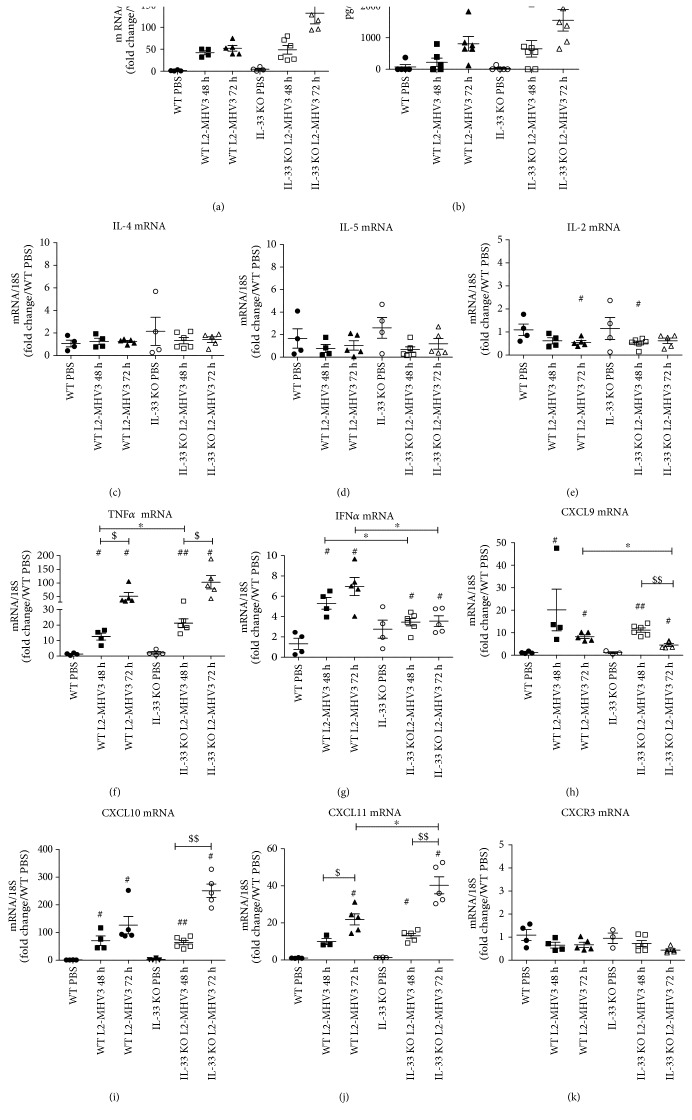
Expression of TH1/TH2 cytokines and chemokines in WT and IL-33 KO mice during L2-MHV3 hepatitis. Total liver RNA was extracted from WT or IL-33 KO mice and control (PBS) or L2-MHV3-infected mice (48 h and 72 h PI) and tested by RT-qPCR for (a) IL-6, (c) IL-4, (d) IL-5, (e) IL-2, (f) TNF*α*, (g) IFN*γ*, (h) CXCL9, (i) CXCL10, (j) CXCL11, and (k) CXCR3. The mean expression of the WT PBS mice was used as a control and arbitrarily considered as 1 AU (arbitrary unit) which served as a reference for fold change in other conditions. (b) Sera from WT or IL-33 KO mice (PBS) or L2-MHV3-infected mice (48 h and 72 h PI) were quantified for IL-6 expression. Statistical analyses were done according to the Mann-Whitney *U* test. # represents significant difference with WT PBS mice, ^∗^ represents significant difference between WT and IL-33 KO mice, and $ represents significant difference between two conditions in the same background mice. ^#^*p* < 0.05; ^##^*p* < 0.01; and ^###^*p* < 0.001.

Finally, we looked at the signature of cytokine expression in IL-33 KO and WT mice to ascertain the exacerbated liver injury in IL-33-deficient mice. The mRNA expression of IL-6 was significantly increased at 48 h and 72 h PI in WT and IL-33 KO mice compared to that in PBS control, while IL-6 expression was significantly upregulated in IL-33 KO mice compared to that in WT mice at only 72 h PI (mean 52 versus 116 AU) (Figure 7(a)). Interestingly, the same profile of IL-6 induction was confirmed at the protein level in the mouse sera (Figure 7(b)). The liver transcriptional expression of classical TH2 cytokines (IL-4, IL-5, and IL-13) was not varied in WT and IL-33 KO mice following L2-MHV3 hepatitis compared to that in PBS control mice (Figures 7(c) and 7(d) and data not shown). Regarding TH1 cytokines, IL-2 was not induced in WT or IL-33 KO mice after L2-MHV3 infection at mRNA level in the liver (Figure 7(e)), neither at protein level in mouse sera (data not shown). The mRNA expression of TNF*α* revealed a strong induction during L2-MHV3 infection in WT and IL-33 KO mice, although enhanced expression was found in IL-33 KO mice especially at 48 h PI (Figure 7(f)). A significant induction of IFN*γ* was found in WT mice (3.6 and 4.2 at 48 h and 72 h PI), whereas IFN*γ* was significantly decreased in IL-33 KO mice (1.4 and 1.9 at 48 h and 72 h PI) compared to that in WT mice at the same time PI (Figure 7(g)). It has to be noted that the basal expression level of IFN*γ* was higher in the IL-33 KO mice compared to that in WT mice (2.6-fold) indicating that IL-33 KO mice were TH1 biased. Nonetheless, no induction was observed during MHV3 infection in IL-33 KO background. We then measured the IFN-induced CXCL9, CXCL10, and CXCL11 chemokine expressions. The CXCL9 was significantly induced at 48 h PI in WT and IL-33 KO mice at the same level followed by a significant decrease in expression at 72 h PI especially in IL-33 KO mice (Figure 7(h)). The CXCL10 and CXCL11 were significantly induced during L2-MHV3 infection in both WT and IL-33 KO mice with an exacerbated manner especially at 72 h PI in IL-33 KO context (Figures 7(i) and 7(j)). Because these IFN*γ*-induced chemokines are known to exert their biological activity via interaction with CXCR3 on TH1 CD4^+^ T cells and CD8^+^ effector T cells, the expression of CXCR3 was measured which showed a global decrease during L2-MHV3 infection in WT and IL-33 KO mice (Figure 7(j)), in correlation with T lymphocyte depletion as observed previously (Figure 4(d)).

## 4. Discussion

IL-33 has been shown to be upregulated in human and murine acute and chronic hepatitis [3, 13, 14, 16, 26]. Recently, we demonstrated that the expression of IL-33 was strongly induced in the nuclei of hepatocytes in a model of viral acute hepatitis induced by the pathogenic mouse hepatitis virus L2-MHV3 [20]. Here, we demonstrated the effect of endogenous IL-33 during L2-MHV3 infection in mice by using IL-33-deficient mice (IL-33 KO).

We observed the protective effect of IL-33 in this acute hepatitis model demonstrated by increased liver injury, weight loss, and the earlier death of the IL-33 KO mice. These could not be explained by higher sensibility of the IL-33 KO mouse strain as the expression of the basic receptors described to be involved in the infectivity such as CEACAM [27], TLR2 [22, 28], and TLR3 [29] receptors were expressed at a comparable level in WT and IL-33 KO mice. A global decrease in liver infiltrate cell number was observed, especially for the immune cell populations of B lymphocytes, T lymphocytes, NKT cells, and macrophages in the WT mice but more importantly in the IL-33 KO mice following L2-MHV3 infection. This decreased immune cell infiltration could be explained by direct virus targeting (i.e., for B and T lymphocytes [30] or macrophages [19]) either by the AICD (activation-induced cell death) for NKT cells or by the lack of survival/proliferation signal as shown for NKT cells which can be directly targeted by IL-33 to increase their number [31]. Interestingly, we observed a dramatic decrease in dendritic cell population at 48 h PI only in the IL-33 KO mice while their number remained stable in the WT mice. It is plausible that IL-33 is critical for dendritic cell proliferation as shown in a model of LCMV-induced hepatitis [17].

In contrast, an increase in the number of neutrophils was observed in IL-33 KO mice after L2-MHV3 hepatitis. It was surprising as it has been shown that IL-33 can drive the priming and the migration of neutrophils in different models in vivo [32, 33]. These authors demonstrated that IL-33 could have, indeed, not only a direct effect on neutrophil recruitment but also an indirect effect by creating an in vivo inflammatory microenvironment that promotes neutrophil priming and recruitment. This was related particularly to the induction of TNF*α*, CXCL1, and CXCL2 expressions in in situ macrophages, fibroblast-like cells, or vessel endothelial cells. Despite the absence of IL-33 and macrophages, these cytokines were over induced by an alternative mechanism in L2-MHV3-infected IL-33 KO mice, in association with other neutrophil-related chemokines such as CXCL5, CCL2, and CCL6, which could be responsible for massive recruitment of neutrophils as observed at 48 h PI. At 72 h PI, we observed a complete disappearance of neutrophils in the liver whereas an enhanced expression of the neutrophil-related chemokines was measured at RNA level in IL-33 KO mice. The fall in neutrophil number could be explained by the short lifespan of the neutrophils after their activation in vivo [34]. The over induction of CXC chemokines could be due to the absence of a negative feedback mechanism driven by CXCR2 expressed on myeloid cells, involved in the regulation of these chemokines' expression as it has been shown in a model of I/R or in CXCR2 KO mice [35, 36]. The ELR-positive CXC chemokines are the key to the pathophysiology of I/R injury, serving as primary chemoattractants that mediate neutrophil recruitment, resulting in subsequent neutrophil-dependent liver injury. In order to demonstrate the direct deleterious effect of this massive neutrophil recruitment in L2-MHV3-infected IL-33 KO mice, it would be interesting to test in vivo neutrophil depletion in this context.

The transcriptomic profile of 284 genes involved in inflammation, cell signaling, and remodeling showed that the expression of IL-1 family members, IL-1*α*, IL-1*β*, and IL-18, was not varied between WT and IL-33 KO mice following L2-MHV3 hepatitis. No difference was observed between the two mouse strains in the ability to drive rather a TH1 or TH2 immune response as the classical cytokines to do so (IL-4, IL-5, IL-13, or IL-2) were not significantly induced at any time of infection. In the liver, IL-6 and TNF*α* are pleiotropic cytokines having the ability to induce cellular responses such as proliferation, inducing hepatocyte multiplication, and liver regeneration but, on the other hand, mediate inflammatory and acute phase liver cell death responses [37, 38]. Here, we found enhanced expression of these cytokines in IL-33-deficient mice compared to those in WT mice. These findings suggest that the increased liver injury in IL-33 KO mice was related with higher expression of IL-6 and TNF*α*. These proinflammatory cytokines were previously described to be induced by MHV3 through TLR2 on macrophages [22]. As the number of liver-infiltrated macrophages was significantly decreased in IL-33 KO mice but in parallel upregulated TLR2 expression was found, it can be explained that the main source of these cytokines might not be macrophages but rather other immune cell population like T lymphocytes or neutrophils. The expression of IFN*γ* increased after the L2-MHV3 infection in WT mice, whereas less profound induction was observed in IL-33 KO mice. In concomitance, the direct link between IL-33 and IFN*γ* upregulation was demonstrated in vivo in viral liver injury model or in vitro studies [17, 31]. These previous studies pointed that IL-33 induced IFN*γ* production not only via iNKT/NK cells but also by *γδ*T cells. The absence of IFN*γ* induction in IL-33 KO mice could be explained partially by the reduction of these cells in IL-33 KO mice during early infection at 48 h PI and reconstitution of NK or NKT cells at 72 h of PI. The CXCL9, CXCL10, and CXCL11 are described to be IFN*γ*-induced chemokines. Nonetheless, the absence of IFN*γ* induction in IL-33 KO and the expressions of CXCL10 and CXCL11 were strongly upregulated during L2-MHV3 infection especially in IL-33 KO background at 72 h PI. We postulate therefore that the induction of these chemokines could be dependent on IFN-*β* in combination with TNF*α* as it has been recently demonstrated in vitro [39]. The CXCL9, as the third member of this family, did not exhibit the same profile expression since it was significantly induced at 48 h PI in WT and IL-33 KO mice by a significant decrease in its expression at 72 h PI in IL-33 KO mice. This suggests that its expression might be more dependent on IFN*γ* than CXCL10 and CXCL11. The CXCL9, CXCL10, and CXCL11 exert their biological function by binding to their specific receptor CXCR3 present on activated T lymphocytes. The observation of decreased CXCR3 expression combined with the fall of lymphocyte number in the liver during hepatitis may explain that despite their induction in IL-33 KO context, these chemokines would not be able to interact with their target cells.

## 5. Conclusion

The alarmin IL-33 has been described to be upregulated in human and murine viral hepatitis. Here, by infecting IL-33-deficient mice (IL-33 KO) with the pathogenic murine hepatitis virus 3 (L2-MHV3), we demonstrated that endogenous IL-33 had multifaceted functions in the regulation of the immune response. Indeed, IL-33 exhibited a hepatoprotective effect by inducting IFN*γ* expression, modulating inflammatory chemokine expression (TNF*α*, IL-6), supporting liver immune cell survival, and regulating neutrophils homing in the liver.

## Supplementary Material

Supplemental Figure 1. False color heat map expression analysis of the up- and down-regulated genes in WT and IL-33 KO mice following L2-MHV3 induced hepatitis. A pool of mRNA, constituted of at least 5 mice by condition, was tested by RT-qPCR for the expression of 344 genes involved in inflammation and cell signaling. The WT PBS condition served as the baseline value. Data processing and gene filtration was carried out using the AMEN suite of tools [25]. The resulting genes were classified into 13 expression patterns (termed P1-P13) using the k-means algorithm bioinformatics analysis and reported in a false-color heat map. Each column corresponds to a sample. Each line represents a gene and its log2-transformed fold-change value. A color scale is shown for log2-transformed fold-change values at the bottom: from blue (low fold-change) to red (high fold-change). Supplemental Table 1: Sequences of specific primers used for RT-QPCR. Supplemental Table 2: Differential expression of 288 genes quantified in the six different conditions (WT PBS vs WT L2-MHV3 48h PI vs WT L2-MHV3 72hPI vs IL-33 KO PBS vs IL-33 KO L2-MHV3 48h PI vs IL-33 KO L2-MHV3 72h PI) and classified in 13 patterns using k-means algorithm bioinformatics analysis. Supplemental Table 3: Highlight on differential expression profiles of neutrophil-related genes quantified in the six different conditions (WT PBS vs WT L2-MHV3 48h PI vs WT L2-MHV3 72hPI vs IL-33 KO PBS vs IL-33 KO L2-MHV3 48h PI vs IL-33 KO L2-MHV3 72h PI).

## Abbreviations

ALT: Alanine aminotransferase
AST: Aspartate aminotransferase
i.v: Intravenous
IL-33: Interleukin 33
KO: Knockout
LL: Liver leukocytes
MHV: Mouse hepatitis virus
MOI: Multiplicity of infection
PI: Post infection
PMH: Primary mouse hepatocytes.
