# Supplementary material for: Endogenous IL-33 Deficiency Exacerbates Liver Injury and Increases Hepatic Influx of Neutrophils in Acute Murine Viral Hepatitis
Source: Mediators Inflamm. 2017 May 4;2017:1359064. doi: 10.1155/2017/1359064 (PMC5457781; doi:10.1155/2017/1359064)
Supplement: Supplementary file 1 — Supplemental Figure 1. False color heat map expression analysis of the up- and down-regulated genes in WT and IL-33 KO mice following L2-MHV3 induced hepatitis. A pool of mRNA, constituted of at least 5 mice by condition, was tested by RT-qPCR for the expression of 344 genes involved in inflammation and cell signaling. The WT PBS condition served as the baseline value. Data processing and gene filtration was carried out using the AMEN suite of tools [25]. The resulting genes were classified into 13 expression patterns (termed P1-P13) using the k-means algorithm bioinformatics analysis and reported in a false-color heat map. Each column corresponds to a sample. Each line represents a gene and its log2-transformed fold-change value. A color scale is shown for log2-transformed fold-change values at the bottom: from blue (low fold-change) to red (high fold-change). Supplemental Table 1: Sequences of specific primers used for RT-QPCR. Supplemental Table 2: Differential expression of 288 genes quantified in the six different conditions (WT PBS vs WT L2-MHV3 48h PI vs WT L2-MHV3 72hPI vs IL-33 KO PBS vs IL-33 KO L2-MHV3 48h PI vs IL-33 KO L2-MHV3 72h PI) and classified in 13 patterns using k-means algorithm bioinformatics analysis. Supplemental Table 3: Highlight on differential expression profiles of neutrophil-related genes quantified in the six different conditions (WT PBS vs WT L2-MHV3 48h PI vs WT L2-MHV3 72hPI vs IL-33 KO PBS vs IL-33 KO L2-MHV3 48h PI vs IL-33 KO L2-MHV3 72h PI). [file 1359064.f1.pdf]

Supplemental figure 1

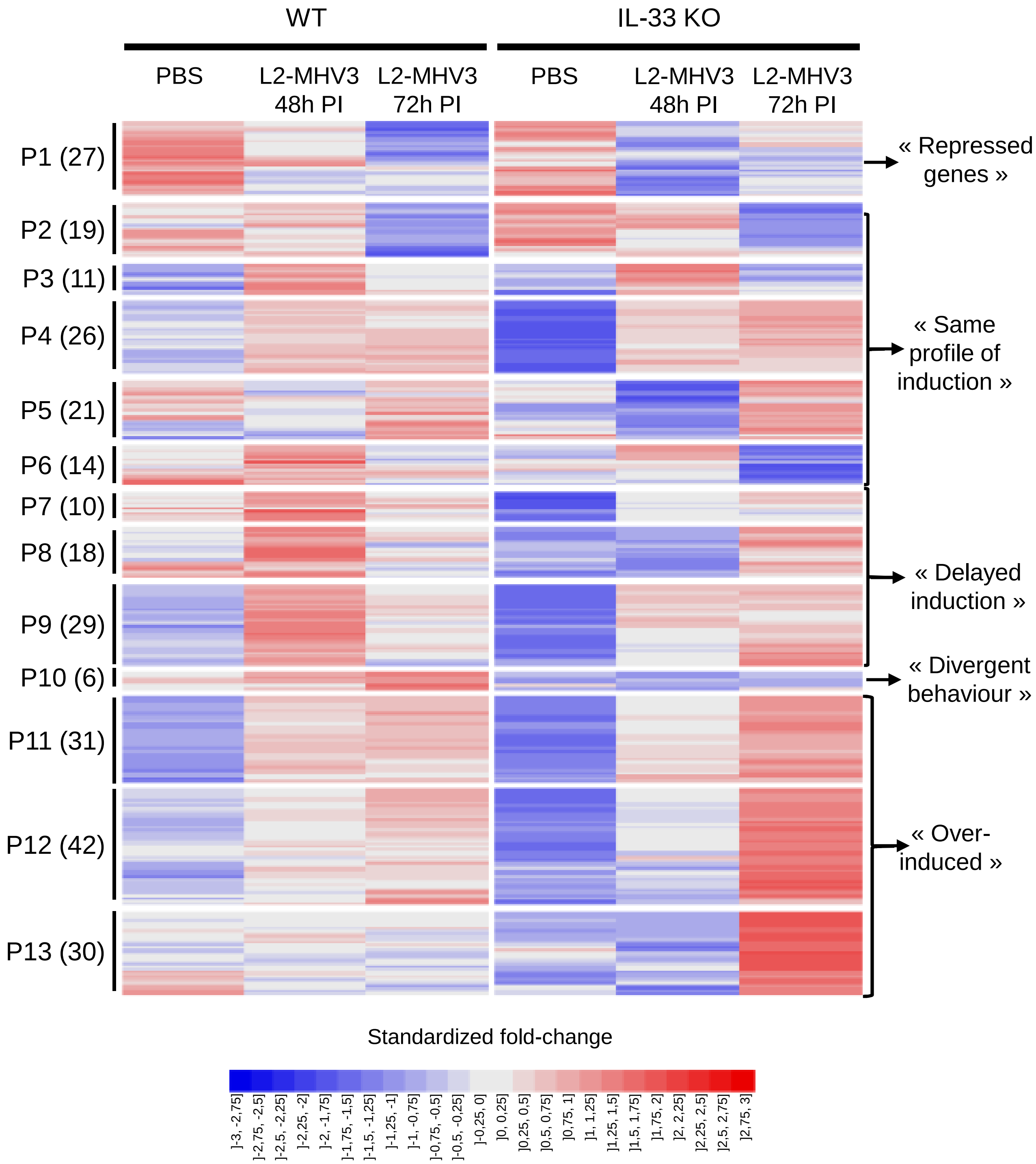

Supplemental figure 1. False color heat map expression analysis of the up- and down-regulated genes in WT and IL-33 KO mice following L2-MHV3 induced hepatitis. A pool of mRNA, constituted of at least 5 mice by condition, was tested by RT-qPCR for the expression of 344 genes involved in inflammation and cell signaling. The WT PBS condition served as the baseline value. Data processing and gene filtration was carried out using the AMEN suite of tools [25]. The resulting genes were classified into 13 expression patterns (termed P1-P13) using the k-means algorithm bioinformatics analysis and reported in a false-color heat map. Each column corresponds to a sample. Each line represents a gene and its log2-transformed fold-change value. A color scale is shown for log2-transformed fold-change values at the bottom: from blue (low fold-change) to red (high fold-change).

Supplemental table 1 : Sequences of specific primers used for RT-QPCR.

Target genes	Forward primer	Reward primer
ARG1	5'-GCA GAG GTC CAG AAG AAT	5'-GTT GCC CAT GCA GAT TCC
CCL2	5'-GTC CCT GTC ATG CTT CTG G	5'-TGC TGG TGA TCC TCT TGT AGC
CCL6	5'-TCT TTA TCC TTG TGG CTG TCC	5'-CTT TTA CAT GGG ATC TGT GTG G
CEACAM1	5' -CTGCCCCTGGCGCTTGGA	5' -TACATGAAATCGCACAGTCGC
CXCL1	5'-CGC CTA TCG CCA ATG AGC	5'-GAA CCA AGG GAG CTT CAG
CXCL2	5'-GAA CTG CGC TGT CAA TGC	5'-GGG CTT CAG GGT CAA GG
CXCL3	5'	
CXCL5	5'-CGC TAA TTT GGA GGT GAT CC	5'-AAC ACT GGC CGT TCT TTC C
CXCL9	5'-CTC CTG CAT CAG CAC CAG	5'-TCT TCA CAT TTG CCG AGT CC
CXCL10	5'-GCT GCC GTC ATT TTC TGC	5'-ACG TGG GCA GGA TAG GC
CXCL11	5'-ACA GCT GCT CAA GGC TTC	5'-GTC CAG GCA CCT TTG TCG
CXCR1	5'-GTC CCA CAT ATT TGG CTT CC	5'-TGT AGG GCA GAC AAC AGA GC
CXCR2	5'-GAG GGT CGT ACT GCG TAT	5'-GGT CTC CTT GAT CAG CTT GG
CXCR3	5'-GTT CTG CTG GTC TCC AGA GG	5'-CTC CCA CAA AGG CAT AGA GC
IFN β	5'-CAG CTC CAG CTC CAA GAA AG	5'-ACC CAG TGC TGG AGA AAT TG
IFN γ	5'-AAG ACA ATC AGG CCA TCA GC	5'-CAG CAG CGA CTC CTT TTC C
IL-2	5'-GAA GAT GAA CTT GGA CCT CTG	5'-AGG GCT TGT TGA GAT GAT GC
IL-4	5'-GGC TTC CAA GGT GCT TCG	5'-GGA CTT GGA CTC ATT CAT GG
IL-6	5'-CGA TGA TGC ACT TGC AGA	5'-CTC TGA AGG ACT CTG GCT TTG
IL-33	5'-ATGGGAAAGAAGCTGATGGTG	5'-CCGAGGACTTTTTGTGAAG
MHV3	5'-TGGAAGGTCTGCACCTGCTA	5'-TTTGGCCCACGGGATTG
TLR2	5'-AGG GAC GTT TGC TAT GAT GC	5'-GTT CGT ACT TGC ACC ACT CG
TLR3	5'-CAC GAA CCT GAC AGA ACT CG	5'-CAC TTC GCA ACG CAA GG
TNFα	5'-CGA GTG ACA AGC CTG TAG CC	5'-AGA TAG CAA ATC GGC TGA CG

Supplemental table 2: Differential expression of 288 genes quantified in the six different conditions (WT PBS vs WT L2-MHV3 48h PI vs WT L2-MHV3 72hPI vs IL-33 KO PBS vs IL-33 KO L2-MHV3 48h PI vs IL-33 KO L2-MHV3 72h PI) and classified in 13 patterns using k-means algorithm bioinformatics analysis.

Gene name	PATTERN	WT PBS	WT L2-MHV3-48h	WT L2-MHV3-72h	IL-33KO PBS	IL-33KO L2-MHV3-48h	IL-33KO L2-MHV3-72h
AHR	1	1,00	0,61	0,33	0,68	0,57	0,52
BMP7	1	1,00	0,54	0,25	1,38	0,49	0,59
GDF2	1	1,00	0,26	0,36	0,54	0,18	0,41
BTLA	1	1,00	1,22	0,40	0,51	0,20	0,34
CCL24	1	1,01	0,94	0,34	1,59	0,66	0,89
CCL27	1	1,00	0,46	0,33	1,05	0,23	0,54
CCR10	1	1,00	0,40	0,12	0,65	0,23	0,19
CD163	1	0,90	0,48	0,22	0,90	0,22	0,56
CD209	1	1,00	0,84	0,12	1,03	0,39	0,71
CNTFR	1	1,00	0,53	0,31	0,69	0,26	0,78
CX3CL1	1	1,01	0,55	0,35	0,52	0,28	0,67
CXCL12	1	1,01	0,36	0,45	0,61	0,20	0,47
CXCR3	1	1,00	0,72	0,42	0,61	0,54	0,42
GFM1	1	1,00	0,54	0,60	1,34	0,46	0,61
FGF1	1	1,00	0,47	0,59	0,81	0,37	0,43
GPT	1	1,01	0,50	0,69	1,72	0,39	0,28
HLX	1	1,00	0,67	0,36	0,76	0,37	0,60
LIFR	1	1,01	0,29	0,31	0,72	0,24	0,32
MRC1	1	1,00	0,54	0,55	0,78	0,29	0,60
PIAS3	1	1,01	1,06	0,49	1,05	0,56	0,56
PTH1R	1	1,00	0,42	0,27	1,22	0,39	0,39
RORC	1	1,00	0,93	0,47	0,63	0,39	0,53
SCARF2	1	1,00	0,83	0,70	1,42	0,50	1,02
TNFRSF19	1	1,00	0,18	0,06	0,63	0,08	0,11
NTRK2	1	1,00	0,20	0,11	2,37	0,04	0,19
VEGFA	1	1,00	0,75	0,87	1,11	0,48	0,68
VEGFB	1	1,00	0,76	0,34	1,23	0,50	0,82
APOE	2	1,01	0,73	0,51	1,05	0,68	0,49
TNFSF13	2	1,00	1,76	0,44	2,56	2,40	0,42
CD276	2	1,00	0,57	0,25	0,98	0,77	0,50
CD4	2	1,00	0,82	0,22	1,81	0,56	0,26
CSF1R	2	1,00	0,61	0,25	0,99	0,49	0,17
KIT	2	1,00	0,92	0,24	1,17	1,24	0,29
EGFR	2	1,00	1,17	0,56	1,53	1,31	0,40
FOXP3	2	1,00	6,00	0,60	3,51	5,65	0,55
IFNAR1	2	1,00	0,80	0,68	1,41	1,34	0,66
CHUK	2	1,01	1,25	0,66	2,00	1,14	0,44
IL18	2	1,00	0,82	0,53	1,15	0,60	0,48
IL21R	2	1,01	1,54	0,02	0,57	1,42	0,76
IL9	2	1,00	4,05	0,01	0,45	2,18	3,01

Gene name	PATTERN	WT PBS	WT L2-MHV3-48h	WT L2-MHV3-72h	IL-33KO PBS	IL-33KO L2-MHV3-48h	IL-33KO L2-MHV3-72h
LIFR	2	1,00	0,92	0,37	2,99	0,76	0,31
SERPINF1	2	1,00	0,43	0,17	0,84	0,33	0,15
STAT5A	2	1,00	1,67	0,56	2,08	1,44	0,56
STAT5B	2	1,00	1,20	0,19	2,20	0,98	0,14
TIRAP	2	1,00	1,61	0,65	2,37	2,68	0,41
NTRK1	2	1,01	0,40	0,04	0,29	0,26	0,14
FLT3LG	3	1,00	2,37	1,71	1,44	1,87	1,67
IKBKG	3	1,00	2,32	1,25	1,42	2,21	0,97
IL1B	3	1,00	2,71	1,99	0,64	2,54	1,41
MYD88	3	1,00	2,79	1,92	1,09	5,08	1,36
NFKB1	3	1,00	1,23	0,94	0,70	1,60	0,65
OSM	3	1,01	10,38	5,56	0,20	8,09	1,01
IL27	3	1,01	8,85	3,28	1,17	6,31	2,10
CD180	3	1,01	3,13	1,89	1,24	4,52	0,89
TBK1	3	1,01	4,88	2,08	1,84	4,77	1,47
TNFRSF1A	3	1,00	2,97	1,50	1,19	3,22	1,03
EDA2R	3	1,01	9,71	2,43	1,16	9,31	2,23
ALPL	4	1,00	2,67	2,13	0,41	2,50	3,87
TNFSF13B	4	1,00	1,42	0,96	0,29	1,65	2,80
CCL12	4	1,01	61,41	50,42	0,18	38,89	65,72
CCL7	4	1,00	48,95	42,10	0,07	34,86	97,04
CCR1	4	1,00	5,91	5,50	0,22	5,54	10,61
LY75	4	1,00	1,34	1,06	0,37	1,31	1,57
CXCR1	4	1,00	3,24	3,00	0,22	2,07	2,04
GCNT1	4	1,00	1,56	1,70	0,78	1,83	1,47
IFNG	4	1,00	3,66	4,22	0,57	1,46	1,94
IL10	4	1,00	11,85	12,14	0,33	5,65	10,26
IL18RAP	4	1,00	1,93	3,00	0,31	2,22	3,36
IL36G	4	1,00	7,98	5,82	0,30	6,14	7,88
IL1R1	4	1,00	3,29	3,98	0,25	1,52	5,69
IFNLR1	4	1,00	2,26	2,17	0,27	1,79	2,86
IL2RB	4	1,00	1,33	1,61	0,46	1,02	1,41
IL2RG	4	1,00	1,62	1,49	0,49	1,73	1,41
CSF2RB	4	1,00	2,11	1,58	0,53	1,65	2,55
LBP	4	1,00	2,83	2,45	0,23	2,21	2,27
CCL3	4	1,00	11,79	23,72	0,16	8,74	8,73
S100A8	4	1,00	9,18	12,53	0,08	8,07	13,53
S100a8	4	1,00	7,83	8,29	0,13	9,91	14,47
S100A9	4	1,01	5,46	4,97	0,11	7,08	10,05
S100A9	4	1,00	6,40	4,54	0,09	6,14	9,84
SAA1	4	1,00	5,72	10,97	0,01	6,26	12,69
SAA3P	4	1,00	10,87	21,31	0,01	12,57	28,37
SOCS3	4	1,00	3,13	5,59	0,22	3,50	8,43

Gene name	PATTERN	WT PBS	WT L2-MHV3-48h	WT L2-MHV3-72h	IL-33KO PBS	IL-33KO L2-MHV3-48h	IL-33KO L2-MHV3-72h
BMP5	5	1,00	0,34	0,45	0,59	0,23	1,01
CD36	5	1,00	0,66	1,14	0,78	0,34	1,33
S1PR1	5	1,01	1,20	2,17	1,80	1,26	1,99
FGFR2	5	1,00	1,23	1,81	1,47	0,65	1,64
GOT1	5	1,00	1,69	3,37	1,54	0,76	2,86
GOT1	5	1,00	1,54	3,50	1,30	0,58	3,26
GPT2	5	1,00	0,84	1,02	0,71	0,37	0,84
IL17RB	5	1,00	0,65	1,00	0,51	0,54	1,19
IL27RA	5	1,00	0,67	0,84	0,55	0,48	0,97
IL6R	5	1,00	0,90	1,22	0,50	0,46	1,47
IL6R	5	1,00	0,88	1,09	0,55	0,48	1,28
LRP5	5	1,00	1,22	2,59	1,27	0,91	3,41
LRP6	5	1,01	0,55	0,85	0,73	0,27	0,51
LRP6	5	1,00	0,71	2,62	1,15	0,55	2,61
PIAS2	5	1,00	0,65	1,12	0,69	0,33	1,83
SMAD7	5	1,01	0,71	1,54	1,75	0,79	1,18
SOCS4	5	1,00	1,11	1,04	1,03	0,53	1,16
TLR5	5	1,00	0,50	0,94	0,80	0,47	1,06
TOLLIP	5	1,00	0,60	0,64	0,54	0,40	0,92
VEGFA	5	1,00	0,74	0,92	0,90	0,47	1,08
WNT5B	5	1,00	0,73	1,73	0,57	0,51	1,45
CXCL13	6	1,00	0,48	0,08	0,11	0,18	0,13
EBI3	6	1,00	1,28	0,64	1,02	1,30	0,46
FGF10	6	1,24	5,11	1,19	1,23	1,24	0,77
FGF3	6	1,00	0,75	0,67	0,30	0,51	0,10
IKZF2	6	1,00	1,31	0,67	0,60	1,49	0,37
IKZF2	6	1,00	1,55	0,81	0,68	1,37	0,57
IL6R	6	1,00	0,66	0,51	0,45	0,36	0,28
CXCR2	6	1,02	4,65	2,32	2,05	2,06	0,15
RPS18	6	1,00	1,02	0,87	0,96	0,96	0,26
TBATA	6	1,00	1,53	0,88	0,85	1,69	0,49
TEK	6	1,01	0,53	0,64	1,18	0,41	0,10
TLR6	6	1,09	2,98	1,14	0,49	2,18	0,21
TSLP	6	1,00	1,32	1,29	0,55	0,76	0,31
XCR1	6	1,01	1,00	0,44	0,47	0,55	0,22
ANG	7	1,00	1,28	0,66	0,43	0,75	0,60
ICOSLG	7	1,00	1,50	0,82	0,41	0,78	0,90
CCR8	7	1,00	2,76	0,68	0,03	0,25	1,22
CD86	7	1,00	1,58	1,01	0,47	0,85	0,84
CD86	7	1,00	1,73	1,34	0,49	0,98	1,39
CRP	7	1,00	1,18	1,04	0,47	0,98	1,04
CXCL9	7	1,00	3,31	1,00	0,09	0,94	1,75
IL23R	7	1,06	5,97	0,98	0,37	1,12	0,87

Gene name	PATTERN	WT PBS	WT L2-MHV3-48h	WT L2-MHV3-72h	IL-33KO PBS	IL-33KO L2-MHV3-48h	IL-33KO L2-MHV3-72h
MARCO	7	1,00	0,49	0,79	0,14	0,37	0,56
TLR1	7	1,00	1,49	1,40	0,32	0,95	0,93
CCR7	8	1,00	3,36	1,09	0,57	0,45	1,24
CD8B	8	1,00	1,54	0,71	0,39	0,50	0,89
ERBB2	8	1,00	0,72	0,49	0,50	0,38	0,80
IL31RA	8	1,00	1,90	1,34	0,72	0,60	2,31
IL15RA	8	1,00	0,83	0,63	0,45	0,43	0,84
IL7R	8	1,01	2,49	1,49	0,56	0,79	2,32
CCL22	8	1,00	2,38	1,13	0,56	0,63	2,03
MMP2	8	1,01	2,11	1,56	1,13	0,76	1,51
SP7	8	1,06	0,91	0,31	0,18	0,09	1,30
TNFRSF4	8	1,00	2,58	0,96	0,99	1,10	2,24
RELT	8	1,00	1,27	0,78	0,48	0,69	1,14
STAT4	8	1,00	3,42	1,50	0,70	0,70	1,62
STAT4	8	1,00	4,12	1,46	0,60	0,46	0,95
TGFB3	8	1,00	1,95	0,51	0,62	0,57	2,55
THY1	8	1,00	1,08	0,60	0,47	0,49	0,69
TLR7	8	1,00	2,14	1,76	0,33	0,49	2,22
LTB	8	1,00	1,87	1,14	0,57	0,73	1,28
FIGF	8	1,00	4,22	1,51	0,86	0,62	2,08
ACVRL1	9	1,00	2,23	1,73	0,74	2,09	1,55
CD274	9	1,00	19,47	7,49	0,32	8,99	9,68
CCR2	9	1,01	2,02	1,61	0,79	1,43	1,44
CCR5	9	1,00	2,64	1,43	0,80	1,33	1,61
FCGR3	9	1,00	2,81	1,53	1,18	2,03	2,09
CD40	9	1,00	4,20	2,26	0,55	2,74	2,37
CD68	9	1,00	1,80	1,15	0,64	1,27	2,02
CD80	9	1,00	3,54	1,78	0,57	1,82	2,23
CXCR4	9	1,01	4,51	1,59	0,56	1,87	4,47
NOS3	9	1,00	2,33	1,42	0,59	1,56	3,36
FAS	9	1,00	2,32	1,37	0,64	1,38	1,48
FASLG	9	1,02	2,23	1,67	0,72	1,09	2,03
FPR2	9	1,00	3,25	1,85	0,54	2,08	2,73
TNFRSF18	9	1,00	2,69	1,75	0,44	1,09	2,80
IL10RA	9	1,00	1,85	0,97	0,65	1,44	1,35
IL13	9	1,00	2,96	0,89	0,81	1,66	4,62
IL19	9	1,01	73,58	7,88	0,13	24,73	21,29
IL1A	9	1,00	2,04	1,33	0,56	1,23	1,66
IL21	9	1,01	2,31	1,13	0,96	1,28	2,48
KITLG	9	1,01	2,47	1,67	0,77	1,40	1,76
IFIH1	9	1,00	6,53	3,65	0,81	4,30	5,18
IL-27	9	1,00	7,36	4,80	0,53	3,42	4,70
STAT1	9	1,01	7,25	4,75	0,55	4,22	6,05

Gene name	PATTERN	WT PBS	WT L2-MHV3-48h	WT L2-MHV3-72h	IL-33KO PBS	IL-33KO L2-MHV3-48h	IL-33KO L2-MHV3-72h
TLR4	9	1,00	2,17	1,59	0,90	1,34	1,76
TNFSF10	9	1,00	8,55	2,39	0,63	4,65	2,63
TNFSF10	9	1,00	7,47	2,29	0,35	2,64	1,47
TNFRSF10A	9	1,05	4,89	2,03	0,44	3,62	3,74
XCL1	9	1,01	2,49	1,97	0,52	1,24	2,23
EDA2R	9	1,01	7,69	3,30	0,27	4,45	4,70
ARG1	10	1,00	1,40	2,16	0,72	0,64	1,32
HPRT1	10	0,92	0,94	2,02	1,11	0,64	0,64
IL12A	10	1,01	1,34	1,42	0,64	0,60	0,73
IL18R1	10	1,01	1,48	1,75	0,76	0,66	0,77
IL33	10	1,03	2,49	3,52	0,00	0,01	0,01
IL33	10	1,01	2,58	3,29	0,00	0,01	0,01
ANGPT2	11	1,00	4,19	3,04	0,58	2,52	10,35
ITGB3	11	1,00	3,45	3,80	0,80	2,63	5,06
CCL2	11	1,00	18,90	50,63	0,28	14,15	207,67
CCRL2	11	1,00	11,05	6,97	0,57	8,23	27,60
CD14	11	1,00	23,44	31,79	0,29	13,18	89,70
CXCL1	11	1,00	13,46	18,30	0,19	13,47	72,81
CXCL10	11	1,00	29,06	33,15	0,28	13,56	51,65
CXCL11	11	1,00	11,40	20,85	0,73	5,02	53,80
CXCL14	11	1,00	2,58	2,01	0,70	6,45	14,21
CXCL2	11	1,00	37,29	88,33	0,36	23,59	593,79
GATA2	11	1,01	2,56	2,47	0,71	1,66	3,09
IFNB1	11	1,32	695,50	1465,43	0,71	604,51	3741,26
IL17RA	11	1,00	3,07	3,25	0,79	3,52	5,59
IL18BP	11	1,00	6,99	7,59	0,51	5,88	7,57
IL18RAP	11	1,00	3,05	4,14	0,40	2,17	6,29
IL1R2	11	1,00	16,14	27,06	0,15	12,61	72,95
IL22RA1	11	1,01	3,85	4,51	0,75	2,37	6,28
IL4R	11	1,00	3,12	4,99	1,04	2,55	8,02
IL6	11	1,01	27,66	19,57	1,68	19,98	64,01
LIF	11	1,00	29,12	51,79	0,28	14,96	145,00
CCL4	11	1,00	21,65	28,36	0,28	17,01	45,27
CCL4	11	1,00	19,21	26,97	0,25	13,83	43,60
OSMR	11	1,00	6,90	9,60	0,65	5,97	13,25
SOCS1	11	1,00	138,64	98,10	1,31	180,81	156,12
TLR2	11	1,00	15,78	16,39	0,30	7,61	24,41
TLR3	11	1,01	11,63	6,42	0,77	7,34	15,24
TLR9	11	1,00	9,84	11,40	0,54	6,21	11,96
TNF	11	1,00	8,66	16,31	0,41	7,41	20,33
CRLF2	11	1,00	2,32	3,38	0,85	1,89	2,99
CRLF2	11	1,00	2,26	3,00	1,35	3,36	3,06
THBS1	11	1,00	16,00	8,70	0,64	8,60	49,70

Gene name	PATTERN	WT PBS	WT L2-MHV3-48h	WT L2-MHV3-72h	IL-33KO PBS	IL-33KO L2-MHV3-48h	IL-33KO L2-MHV3-72h
TNFSF9	12	1,01	2,17	2,77	0,78	1,78	8,62
TNFSF9	12	1,01	6,10	16,72	0,13	2,63	34,26
ITGA5	12	1,00	1,36	1,61	0,76	1,30	2,27
ACTB	12	1,01	1,05	1,11	0,65	0,94	1,40
B2M	12	1,00	1,82	2,00	1,04	1,17	3,99
CCL6	12	1,00	2,23	3,06	0,46	1,62	7,55
ITGAM	12	1,01	2,08	6,38	0,19	1,67	8,32
ITGAX	12	1,00	1,80	4,58	0,39	1,38	9,55
SELP	12	1,00	2,52	6,14	0,51	1,89	27,42
CLC	12	1,00	2,27	3,64	0,49	1,22	148,59
CLEC1A	12	1,00	1,51	1,89	0,62	0,89	1,52
CXCL16	12	1,00	1,19	0,98	0,59	0,80	1,80
CXCL5	12	1,01	3,25	3,19	0,13	1,88	25,92
PPBP	12	1,01	1,03	0,82	0,26	1,15	2,80
FGA	12	1,00	1,08	1,77	0,42	1,16	2,79
FGB	12	1,00	1,37	2,24	0,39	1,18	3,26
FGF2	12	1,00	2,74	2,27	1,32	0,89	5,15
FGG	12	1,00	1,01	1,50	0,53	1,73	3,15
GATA3	12	1,01	1,03	1,80	0,54	0,82	1,32
GZMB	12	1,00	11,42	22,71	0,36	5,15	171,54
GSR	12	1,00	1,27	3,37	0,89	1,11	4,66
ICAM1	12	1,00	2,68	6,73	0,23	1,48	8,16
IFNAR2	12	1,00	1,50	1,61	0,76	1,13	2,06
IKBKB	12	1,00	1,15	1,66	0,90	0,84	2,23
IL10RB	12	1,00	0,97	1,24	0,74	0,79	1,60
IL12RB2	12	1,01	3,29	2,65	0,62	1,33	27,65
IL13RA1	12	1,00	1,75	1,47	0,53	1,19	3,42
CSF1	12	1,00	1,72	2,88	0,31	1,11	6,76
MMP14	12	1,00	1,22	1,46	0,59	0,89	3,14
TNFRSF11B	12	1,00	1,77	1,77	0,59	1,08	3,36
PDGFRB	12	1,00	2,07	1,88	0,99	1,58	3,47
PDGFRB	12	1,01	1,83	1,67	0,83	1,14	3,65
SELE	12	1,00	3,29	4,44	0,35	2,62	18,86
STAT3	12	1,00	1,80	2,19	0,52	1,03	3,03
TGFBR2	12	1,01	1,26	1,14	0,67	0,91	1,94
TLR7	12	1,00	2,01	1,89	0,99	1,29	2,81
TLR8	12	1,00	2,08	4,98	0,92	1,01	4,62
TNFRSF1B	12	1,00	1,38	1,13	0,58	1,02	1,88
TNFRSF1A	12	1,00	2,18	2,89	0,71	1,32	3,19
TRAF6	12	1,00	1,85	2,59	1,01	1,11	3,85
TNFRSF12A	12	1,01	3,96	6,62	0,57	1,73	22,52
VEGFC	12	1,00	2,34	2,57	1,44	1,76	4,69
BMP10	13	1,01	0,42	0,46	0,43	0,23	1,13

Gene name	PATTERN	WT PBS	WT L2-MHV3-48h	WT L2-MHV3-72h	IL-33KO PBS	IL-33KO L2-MHV3-48h	IL-33KO L2-MHV3-72h
CTSK	13	1,00	1,06	0,78	0,71	0,70	1,81
CCL5	13	1,00	0,27	0,51	0,07	0,12	1,36
CCL9	13	1,01	0,59	0,64	0,62	0,50	1,14
CISH	13	1,00	0,78	0,65	0,76	0,50	1,12
COL1A1	13	1,00	1,18	0,83	0,61	0,71	2,58
SLC6A8	13	1,00	0,74	0,54	1,06	0,68	10,81
PF4	13	1,00	0,83	0,79	0,54	0,51	2,65
EPHB2	13	0,82	1,12	0,97	1,54	0,58	2,62
F3	13	1,00	0,65	0,24	0,64	0,19	1,40
FGFR1	13	1,00	1,05	1,04	0,85	0,83	1,84
HGF	13	1,00	1,06	0,96	0,86	1,13	2,51
IL2	13	1,00	1,22	0,60	0,51	1,14	6,29
IRAK4	13	1,00	0,90	0,82	0,46	0,60	1,30
LEPR	13	1,00	1,34	1,29	0,40	0,52	10,77
LRP5	13	1,00	1,62	1,07	1,90	1,25	25,65
SPP1	13	1,00	1,17	2,54	0,87	1,40	78,24
PDCD1	13	1,00	0,53	0,36	0,07	0,44	6,13
PDGFA	13	1,01	0,89	0,88	1,13	0,75	2,20
PDGFRA	13	1,00	0,97	0,88	0,66	0,62	2,62
PECAM1	13	1,00	0,97	0,80	0,65	0,68	1,56
RORA	13	1,00	1,35	1,36	1,11	0,78	2,23
RUNX2	13	1,01	0,80	0,72	0,42	0,42	1,26
SMAD3	13	1,00	1,15	1,11	0,91	0,86	2,79
SMAD4	13	1,00	0,99	1,01	0,91	0,60	1,60
SOCS6	13	1,00	1,47	1,26	0,90	0,74	2,18
TGFB2	13	1,01	0,40	1,27	0,63	0,33	44,33
TGFBR1	13	1,00	0,48	0,76	0,39	0,53	1,59
CDH5	13	1,00	1,27	0,91	0,78	0,80	1,96
WNT5A	13	1,00	0,89	1,45	0,60	0,58	3,00

Supplemental table 3: Highlight on differential expression profiles of neutrophil-related genes quantified in the six different conditions (WT PBS vs WT L2-MHV3 48h PI vs WT L2-MHV3 72hPI vs IL-33 KO PBS vs IL-33 KO L2-MHV3 48h PI vs IL-33 KO L2-MHV3 72h PI).

Gene name	PATTERN	WT PBS	WT L2-MHV3-48h	WT L2-MHV3-72h	IL-33KO PBS	IL-33KO L2-MHV3-48h	IL-33KO L2-MHV3-72h
CXCR1	4	1,00	3,24	3,00	0,22	2,07	2,04
CXCR2	6	1,02	4,65	2,32	2,05	2,06	0,15
CCL2	11	1,00	18,90	50,63	0,28	14,15	207,67
CXCL1	11	1,00	13,46	18,30	0,19	13,47	72,81
CXCL2	11	1,00	37,29	88,33	0,36	23,59	593,79
CCL6	12	1,00	2,23	3,06	0,46	1,62	7,55
CXCL5	12	1,01	3,25	3,19	0,13	1,88	25,92
